# Comparison of the amorphization ability of two polymorphic forms of olanzapine

**DOI:** 10.1080/07853890.2021.1896100

**Published:** 2021-09-28

**Authors:** Catarina Chendo, Nuno F. da Costa, João F. Pinto, Ana I. Fernandes

**Affiliations:** a iMed.ULisboa, Faculdade de Farmácia, Universidade de Lisboa, Lisboa, Portugal; b Centro de Investigação Interdisciplinar Egas Moniz (CiiEM), Egas Moniz Cooperativa de Ensino Superior, Caparica, Portugal

## Abstract

**Introduction:**

The production of amorphous and co-amorphous (CAM) materials has been used as a procedure to overcome the poor water solubility shown by most of the drugs currently under development [1]. Ball milling has been considered to convert the crystalline state of a substance into its amorphous counterpart [2,3]. At present, the impact of the initial polymorphic form of a drug substance on its final amorphous state upon milling, has not been clearly studied. This work aims to compare the co-amorphization efficiency of two different polymorphic forms of olanzapine (OLZ; a BCS class II drug) using saccharin (SAC) as a co-former.

**Materials and methods:**

OLZ (forms I and II) were the starting polymorphic forms to be milled with SAC (2:1 molar ratio). OLZ form I was used as received while OLZ form II was obtained from crystallisation of OLZ in dichloromethane. Ball milling was performed using 2.5 g of 3.0 mm Ø balls, for 2 h. The conversion of the crystalline states into the amorphous counterpart was monitored by calorimetry (DSC) and diffractometry (XRPD).

**Results:**

The thermograms and the diffractograms obtained (Figure 1) have shown a clear difference between the final products of the two polymorphic forms of OLZ. XRPD peaks were less intense for OLZ form II and most representative of SAC, indicating that OLZ was indeed mostly converted into the amorphous state. On the other hand, when OLZ form I was the starting material, a richer diffractogram resulted, suggesting that a crystalline fraction of OLZ remained in the particles’ network of the final product, a feature also confirmed by DSC. **Discussion and conclusions:** Since OLZ form II has a higher thermodynamic energy than form I, it is not surprising that the former exhibits better amorphization ability. Thus, it is expected that either an increase on milling time, or milling speed, would enhance the co-amorphization of OLZ form I with SAC.

**Figure 1.**
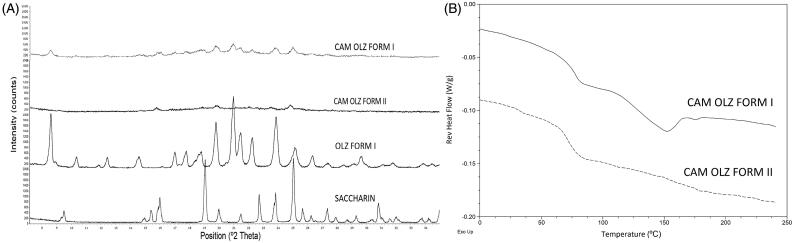
X-ray diffractograms (A) and DSC thermograms (B) of the different molecular entities..
